# Critical commentary on: "Effect of levothyroxine on miscarriage among women with normal thyroid function and thyroid autoimmunity undergoing *In Vitro* fertilization and embryo transfer: a randomized clinical trial"

**DOI:** 10.3389/fendo.2025.1637122

**Published:** 2026-01-07

**Authors:** Yasmin Magdi

**Affiliations:** Al-Yasmeen Fertility and Gynecology Center, Benha, Qalubyia, Egypt

**Keywords:** thyroid, ICSI, levothyroxine, miscarriage, TPOAb, integrity of RCT

## 1 Introduction

I have read the well-conducted randomized controlled study (RCT) published by Wang et al., 2017 in JAMA, known as The Pregnancy Outcomes Study in Euthyroid Women with Thyroid Autoimmunity after Levothyroxine (POSTAL) study with great interest. Authors of the POSTAL study randomized 600 euthyroid women undergoing ICSI with positive anti-thyroperoxidase antibodies (TPOAb) to receive either levothyroxine or no treatment (1). Authors concluded that levothyroxine did not reduce the rate of miscarriage or improve the rates of pregnancy and livebirth.

While reading this interesting RCT, I have identified some concerns that may make the transparency and trustworthiness of the POSTAL study questionable. To assess and address my concerns systematically, I used the Trustworthiness in Randomised Clinical Trials (TRACT) checklist (2).

## 2 TRACT checklist

TRACT checklist has 7 domains with 19 items that are applicable to check trustworthiness of any RCT in medical research (2). These domains are governance, author group, plausibility of intervention usage, timeframe, drop-out rates, baseline characteristics, and outcomes. The items of checklist were rated as either ‘‘no concern’’, ‘‘some concern/no information’’, or ‘‘major concern”. Rating the overall risk of integrity to either low, moderate or high risk depends on the number of domains with concerns.

To re-calculate p-values of summary data of baseline characteristics, ANOVA method was used (3). This method was recommended by many experienced sleuths and TRACT checklist developers (2, 3). To ensure and verify the findings, summery data was sent to an independent statistician to perform the Monte Carlo simulations test, as previously described (4).

The Chinese Clinical Trial Registry (ChiCTR-TRC-13004097) was searched to identify the time of registration. I also searched for any explanation in the final report about any reason or problem declared by the authors that could hinder the process of prospective registration.

To identify retracted studies of author group, I searched Retraction Watch Database and PubMed using each author’s name and institution. I searched PubMed and Embase using the registry number to identify any other presentation of the same RCT and reviewed methods and results for consistency.

Using TRACT checklist (2), five concerns were identified in 5 domains out of seven. Details of the formal assessment using TRACT checklist of the POSTAL study are available online (5). The concerns are summarized as follows:

### 2.1 Governance and trial registration

**Concern:** The POSTAL, according to the Chinese Clinical Trial Registry (ChiCTR-TRC-13004097), was retrospectively registered on December-2013, which is 15 months after the first patient recruitment. The authors noted this delay (as 12 months) in their ESHRE abstract.

Trustworthiness implication: Prospective registration is fundamental step for transparent RCT conduct since 2010 (2). It is crucial to prevent selective reporting of outcomes. Retrospective registration without a declared reason for the delay by the authors and the journal’s acceptance of the exception significantly compromises the transparency of the trial’s governance (6) and raises an initial “major concern” regarding reporting bias.

### 2.2 Author group

**Concern:** The search for retracted studies identified two retractions for a single author in the group. One retracted RCT due to duplicate submission (7) and another retracted abstract of RCT due to data/conclusion errors.

Trustworthiness implication: While not directly related to the POSTAL study’s data, a history of retractions due to critical and ethical issues within the author group is a relevant factor when assessing integrity and adherence to ethical standards.

### 2.3 Baseline characteristics

Concern: Independent statistical re-analysis of the baseline characteristics showed significant statistical imbalances that were not fully reported by the authors.

Recalculation: The re-calculation of p-values found significant differences in serum TSH, total thyroxine, free thyroxine, testosterone, and TPOAb levels.

Author Reporting: The authors only acknowledged the TSH difference, describing it as a “little higher median value”, despite the high significance (P < 0.0001) and large magnitude of the difference.

Monte Carlo Simulation: A more robust Monte Carlo simulation confirmed that the baseline data distribution is unlikely to have resulted from proper randomization (P = 0.000864 and P = 0.0009373).

Trustworthiness implication: Significant imbalances in baseline variables suggest a potential breach of the randomization process, which is a “major concern.” Furthermore, the inconsistent reporting and downplaying of the magnitude of these differences raises concerns about potential data falsification (3) or selective presentation of results.

### 2.4 Timeframe

Concern: The trial timeline for patient enrollment and screening appears to be questionable. Rate of recruitment is inconsistent with the capacity of recruiting center reported in their protocol.

Trustworthiness implication: The speed of patient enrollment is crucial in assessment of the trustworthy of RCTs. Fast rate of recruitment raises concern.

### 2.5 Differences between abstract and main manuscript

Concern: Data of the POSTAL study showed major discrepancies between the manuscript (1) and the ESHRE abstract (8), including flow of participants, duration of patient enrollment and clinical outcomes.

Trustworthiness implication: It is not uncommon for abstracts to contain summarized or preliminary data that may differ from the final published manuscript, especially if there were updates or corrections after the abstract submission. However, significant discrepancies should be clearly explained or justified.

## 3 Discussion

Results from the POSTAL study are warranted to resolve the debate about the effect of positive TPOAb in euthyroid women undergoing IVF-ET on spontaneous miscarriage and clinical outcomes, showing that treatment of Levothyroxine is with no benefit (1). The authors recruited large sample size of patients through a rigorous study design to inform the current practice with valuable insights and conclusions. However, it is crucial to incorporate research integrity screening of RCTs into evidence synthesis to ensure adequate reporting of results and rigorous methodology without significant compromised integrity (9). Formal assessment of the integrity of POSTAL study using the TRACT checklist has identified major concerns regarding retrospective trial registration, baseline imbalances, inappropriate data reporting and timeline discrepancies. These major and multiple concerns in most domains indicate that the overall integrity risk level of the POSTAL study is high, marking it as suspicious.

Compromised RCT integrity puts the patients at risk of receiving ineffective or harmful treatment. Post-publication raised here should be addressed to avoid any potential contamination of the evidence and to ensure the safety of patients (10). The investigators should show a willingness to address these post-publication concerns and share the raw data in a public repository (11). Given that the raised concerns are serious, journals, publishers and institutions have an ethical obligation to the community to go through investigations (11).

The widespread belief that publishing RCTs in high-ranked journals is a marker of trustworthiness is no longer guaranteed. Concerns in this commentary showed that even RCTs published in top ranked journals suffer major challenges regarding inadequate trial registration and inappropriate reporting. Despite the efforts, adherence to ethical obligations of prospective registration and reporting related to ethics and consent remain inadequate (12, 13). The present concerns emphasize the need to ensure stringent governance through harmonizing the process of ethical approval worldwide and linking it to obligatory prospective registration (14).

Inaccuracies and inconsistencies in data reporting presented here are alarming. These concerns emphasize the need for more efforts to promote accurate and transparent reporting of RCTs. Strict adherence to international guidelines and recommendations and deployment of reporting checklists (e.g. CONSORT, GRIPP, etc.) is crucial to avoid inappropriate reporting and ensure transparency and openness (15, 16).

In conclusion, inadequacies in prospective registration and reporting related to ethics remain major challenges in medical research integrity. These inadequacies highlight the greater need for improvement areas in clinical trials including standardization of stringent governance and adherence to reporting guidelines and recommendations to safeguard the integrity of RCTs.
